# Miniaturized Optical Resolution Photoacoustic Microscope Based on a Microelectromechanical Systems Scanning Mirror

**DOI:** 10.3390/mi9060288

**Published:** 2018-06-07

**Authors:** Weizhi Qi, Qian Chen, Heng Guo, Huikai Xie, Lei Xi

**Affiliations:** 1 Department of Biomedical Engineering, Southern University of Science and Technology, Shenzhen 518055, China; qiweizhi@std.uestc.edu.cn (W.Q.); QianChen@std.uestc.edu.cn (Q.C.); guoheng95@std.uestc.edu.cn (H.G.); 2 School of Physics, University of Electronic Science and Technology of China, Chengdu 610054, China; 3 Department of Electrical and Computer Engineering, University of Florida, Gainesville, FL 32611, USA; hkxie@ece.ufl.edu

**Keywords:** photoacoustic, microelectromechanical systems (MEMS), miniaturized microscope

## Abstract

In this paper, we report a miniaturized optical resolution photoacoustic microscopy system based on a microelectromechanical system (MEMS) scanning mirror. A two-dimensional MEMS scanning mirror was used to achieve raster scanning of the excitation optical focus. The wideband photoacoustic signals were detected by a flat ultrasound transducer with a center frequency of 10 MHz and an active area of 2 mm in diameter. The size and weight of this device were 60 mm × 30 mm × 20 mm and 40 g, respectively. We evaluated this system using sharp blades, carbon fibers, and a silver strip target. In vivo experiments of imaging vasculatures in the mouse ear, brain, and human lip were completed to demonstrate its potential for biological and clinical applications.

## 1. Introduction

Optical resolution photoacoustic microscopy (ORPAM) is one of the fastest evolving microscopic imaging techniques [1,2,3,4]. ORPAM uses a highly converging laser beam with an ultrashort pulse duration to generate wideband acoustic waves that can be detected by acoustic transducers [5,6]. It uses intrinsic biological contrast and has a comparative lateral resolution with a deeper penetration depth compared with conventional pure optical microscopic imaging modalities [7]. In a conventional ORPAM, a two-dimensional (2D) motorized mechanical scanner is required to perform raster scanning of the overlapped optical and acoustic focuses to form a three-dimensional (3D) image [8,9,10]. However, improving the temporal resolution and realizing miniaturization of these mechanical-scanning-based ORPAMs are difficult due to the limited moving velocity and bulky size of the scanners. To achieve high-speed imaging and compact configuration, several new scanning mechanisms and scanners have been proposed. Xi et al. developed an intraoperative photoacoustic tomography system with a lateral resolution of up to 0.2 mm based on a microelectromechanical systems (MEMS) scanning mirror and a ring-shaped ultrasound transducer [11]. In addition, a rotary-scanning-based portable ORPAM (pORPAM) has been reported and applied to various biomedical and clinical applications [12,13]. Wang and colleagues used a water-immersible magnetic MEMS mirror with a plate size of 9 mm × 9 mm to simultaneously scan both optical and acoustic focuses for fast brain and single circulating melanoma cells imaging [14,15,16]. As an improvement on previous systems, they reported a handheld ORPAM using a newly developed two-axis magnetic MEMS mirror with an active imaging area of 2.5 mm × 2.5 mm, and dimensions of 80 mm × 115 mm × 150 mm [17]. Wang et al. also reported magnetic MEMS-based all-optical photoacoustic microscopy [18]. Kim et al. introduced a high-speed and high-signal to noise ratio (SNR) photoacoustic microscopy immerged in non-conducting liquid [19], and a polydimethylsiloxane (PDMS)-based two axis MEMS scanner with a size of 15 mm × 15 mm × 15 mm for developing a handheld ORPAM [20,21,22]. Use of a graded-index (GRIN) lens integrated with an image-guided optical fiber bundle, Zemp et al. developed a 500 g, 40 mm × 60 mm portable ORPAM probe with an imaging area of 800 μm [23]. Although these attempts are encouraging, the systems’ inherent limitations are still challenging for clinical applications.

In this study, we used an electrothermal-bimorph-actuation-based MEMS mirror to develop a miniaturized ORPAM probe. The MEMS mirror had a diameter of 2 mm, a resonant frequency of 800 Hz, and a 7° optical scanning angle [24]. The entire probe was 60 mm × 30 mm × 20 mm with a weight of 40 g. Phantom experiments showed that the system has a lateral resolution of 10.4 μm and an active imaging area of 0.9 mm × 0.9 mm. In vivo imaging of a mouse ear, brain, and human lip were performed to highlight the potential biological and clinical applications.

## 2. Materials and Methods

### 2.1. Configurations of the Imaging System and Probe

**Figure 1:**
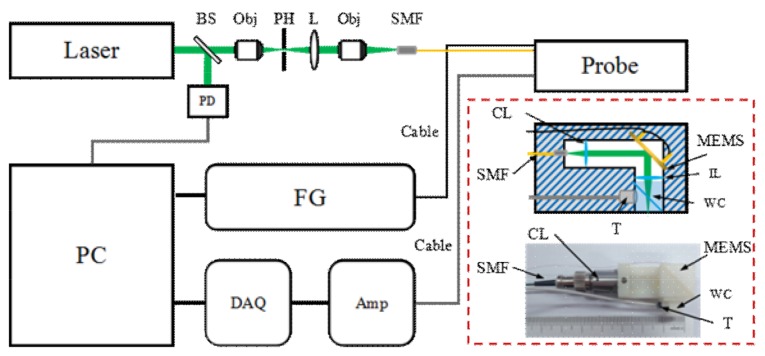
The schematic of the system and imaging probe. The detailed design and photograph of the imaging probe are provided in the dashed red box. The dimensions of the probe were roughly 60 mm × 30 mm × 20 mm. PD: photodiode, Obj: Objective, SMF: single mode fiber, FG: function generator, DAQ: data acquisition card, AMP: amplifier, CL: collimator, T: transducer, L: convex lens, BS: beam splitter, PH: pinhole, PC: personal computer, IL: imaging lens, and WC: water cube.

The illumination of the MEMS-based ORPAM is presented in Figure 1. Laser pulses with a pulse energy of 20 μJ, a 5 ns duration, and a repetition rate of 10 kHz were emitted from a 532 nm Nd:YAG laser (FQS-Y-1-532, Elforlight, Daventry, UK) that was split into two parts through a cover glass. The reflected part was delivered to a photodiode (PD818-BB-21, Newport Corp., Irvine, CA, USA), which was used as the trigger and synchronization signals for MEMS scanning and data acquisition. The transmission part was spatially filtered through a customized spatial filter with a 10× objective (GCO-2112, Daheng Optics, Beijing, China), a 15 μm pinhole, and a Plano-convex lens, and then coupled into a single-mode optical fiber via a space-to-fiber coupler built by an objective (GCO-2112, Daheng Optics) and a customized five-dimensional single mode optical fiber manipulator. The output beam from the optic fiber was collimated by a fiber-compatible collimator (F240FC-532, Thorlabs Inc., Newton, NJ, USA) and then scanned by the MEMS mirror (WM-LS_5, WiO TECH, Wuxi, China). The MEMS was driven by a multifunctional data acquisition card (PCI-6731, National Instrument, Austin, TX, USA) to achieve raster scanning on the surface of an imaging lens (AC080-010-A, Thorlabs Inc., Newton, NJ, USA), using triangle waves for the fast axis and saw-tooth waves for the slow axis. To allow full transmission of the laser pulses and reflection of the generated photoacoustic signals, a water cube including two isosceles right-angle water prisms using a tilted 0.1 mm cover glass was used. The photoacoustic signals were recorded by a small ultrasound transducer (center frequency: 10 MHz, active area: 2 mm in diameter, bandwidth: 60%), amplified by a homemade low noise pre-amplifier at ~66 dB, acquired by a high-speed data acquisition card (PXI-5124, NI, Austin, TX, USA) and stored in a computer for image reconstruction.

### 2.2. Phantom and Animal Experiments

To measure the field of view (FOV) and spatial resolution of the system, a metal strip target with a strip width of 0.1 mm and the edge of a shape surgical blade were imaged. We also performed phantom experiments and in vivo experiments to further assess the system. Multiple 7 μm diameter carbon fibers embedded in a tissue mimicking agarose phantom mixed with India ink and intralipid were imaged to simulate the vasculature in biological tissues. Then mouse ears and brains were imaged to evaluate the system performance for in vivo animals. During the experiment, mice were kept motionless using an isoflurane inhalation anesthetic system and the body temperature was maintained at 37 °C using a temperature control pad (17673, Lankai Inc., Changzhou, China). The ears were gently depilated, then tightly attached to the sealing membrane. For brain imaging, scalps were carefully removed with a sharp blade and skulls remained intact. All mice were kept 24 h after the experiments and no obvious damage were observed in the imaging area. The frame size for the phantom and animal experiments was 500 × 500 pixels. Considering the maximal repetition rate of the laser source, each experiment lasted 28 s. All procedures were approved by the Southern University of Science and Technology.

### 2.3. In Vivo Human Experiments

To demonstrate the clinical potential of this probe, we performed in vivo human oral imaging. During the experiments, the male volunteer sat on a chair and wore protective goggles to avoid potential laser damage to the eyes. The image probe was held and attached to the volunteer’s lower lip. Due to insufficient repetition rate of the laser source and involuntary movement of the human lip, we reduced the frame size (250 × 250 pixels) to improve the imaging speed. A total of 8 s were required to acquire each volume data. After the experiments, the dentist inspected the imaging area and no obvious damage was observed. We obtained consent from the volunteer and all procedures were approved by the Southern University of Science and Technology.

## 3. Results

**Figure 2:**
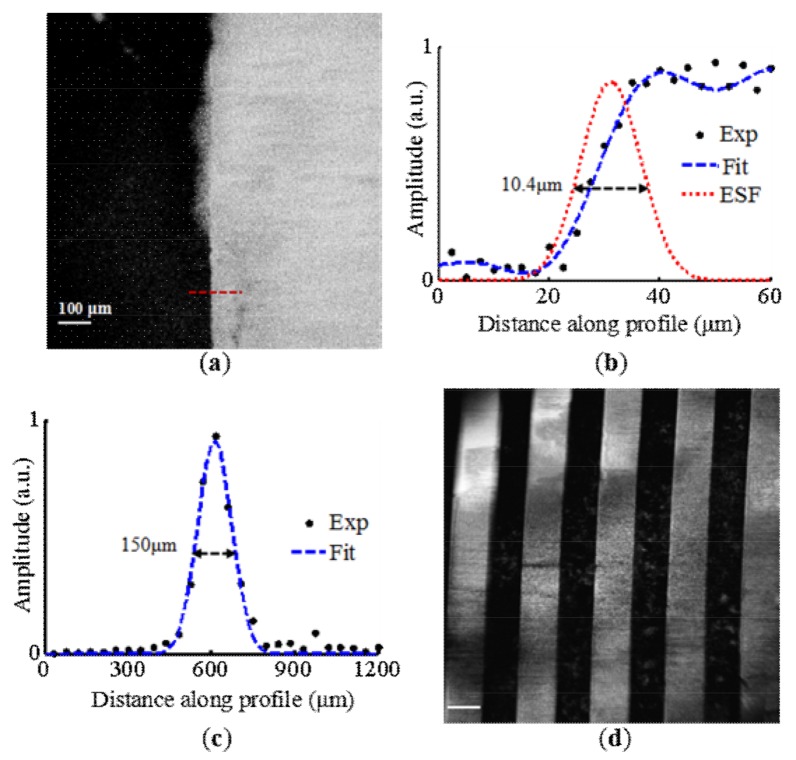
Evaluation of the system performance. (**a**) The maximum amplitude projection (MAP) image of the sharp blade. Estimation of the (**b**) lateral and (**c**) axial resolutions of the system. (**d**) The MAP image of the silver strip target to measure the effective field of view (FOV). Scale bar: 100 μm.

Figure 2a shows the maximum amplitude projection (MAP) image of the sharp blade. To estimate the lateral resolution, a profile along the red dashed line was fitted to obtain the edge spread function (ESF), which was used to derive the line spread function (LSF), as shown in Figure 2b. By calculating the full width-at-half-maximum (FWHM) of the LSF, the lateral resolution was measured as 10.4 μm. As shown in Figure 2c, the FWHM of the Gaussian-fitted axial profile of a typical depth-resolved PA signal was about 150 μm, which represents the axial resolution of the system. Considering that the diameter of the optical focus was 10.4 μm and the energy of the laser pulse after the imaging lenses was 80 nJ, the energy density at the focal plane in the air was 94 mJ/cm^2^. When we assembled the probe, the optical focal plane was adjusted to be approximately l0.25 mm outside the imaging window, leading to a photon energy density of 1 mJ/cm^2^ on the tissue surface during in vivo experiments. Figure 2d shows the MAP image of the silver strip target. There were five strips in the entire imaging area, resulting in an effective area of 0.9 mm × 0.9 mm. In addition, no obvious distortion of the strips was evident over the entire imaging domain.

**Figure 3:**
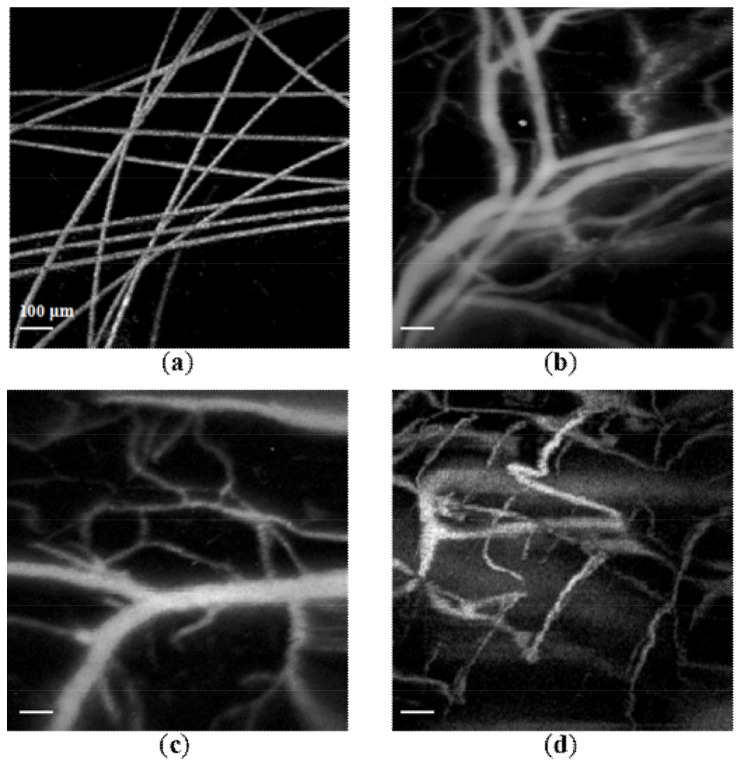
The MAP images of (**a**) carbon fibers, (**b**) blood vessels in a mouse ear and (**c**) brain, and (**d**) vasculature in a human lower lip. The frame size of images in (**a**–**c**) is 500 × 500 pixels, and the image in (**d**) is 250 × 250 pixels. Scale bar: 100 μm.

Figure 3a shows the MAP image of carbon fibers buried in an agarose phantom. We could clearly distinguish each carbon fiber with sufficient contrast and resolution. Figure 3b,c present the MAP images of the vascular networks inside a mouse ear and brain, respectively. Large, medium, and small blood vessels are clearly identified. However, we noticed that the SNR and resolution in Figure 3b is better than in Figure 3c. The major reason for this observation is that the skull of the brain significantly reduces the photoacoustic signals and slightly distorts the optical focus due to optical scattering. Figure 3d shows the MAP image of the human lower lip, in which we observed vasculatures with acceptable image quality. The animal and human experiments suggest that the proposed miniaturized ORPAM probe is applicable for both fundamental and clinical applications.

## 4. Discussion and Conclusions

In this work, we reported a miniature ORPAM imaging probe using a 2D MEMS mirror and a small flat ultrasound transducer. The performance of the system was evaluated with phantom, animal, and human experiments. The MEMS mirror, being small, light, fast, and inexpensive, considerably reduces the probe size. The imaging results suggest that this technique holds potential for both fundamental and clinical applications. For example, in brain surgery, using this probe is feasible to evaluate bypass-grafted blood vessels based on morphological and functional information, such as vessel diameter, blood flow, and oxygen saturation (sO_2_). The other potential application is to detect early-stage oral cancer. By imaging the lips, small blood vessels, and especially capillary loops, can be clearly observed. The morphological changes in these capillary loops are tightly related to the occurrence of oral cancer. However, this probe has some limitations. Firstly, the imaging area is relatively small, which is mainly caused by the limited scanning angle of the MEMS mirror and the probe size. To increase the domain, either the design of MEMS mirror can be optimized to increase the scanning angle, or a new design can be proposed for the optical path. Secondly, the spatial resolution was insufficient to visualize cellar level targets. In theory, the lateral resolution is mainly determined by the numerical aperture (NA) of the focusing lens and the axial resolution depends on the center frequency and bandwidth of the ultrasonic transducer. Hence, we could achieve higher spatial resolution by using a high-NA focusing lens and a high frequency transducer with a broader bandwidth. Thirdly, the imaging speed of the system was limited by the repetition rate of the laser. If a 300 kHz repetition rate laser is applied, we could acquire a large volume data within 1 s, which would mostly eliminate the motion artifacts of in vivo experiments and extend the applications of this probe.
